# An angiotensin-converting enzyme inhibitor modulates stromal-derived factor-1 through CD26/dipeptidyl peptidase IV to inhibit laser-induced choroidal neovascularization

**Published:** 2013-05-29

**Authors:** Hong Li, Yu-sheng Wang

**Affiliations:** 1 Department of Ophthalmology, Eye Institute of Chinese PLA, Xijing Hospital, Fourth Military Medical University, Xi’an, PR China; 2 Department of ophthalmology, General Hospital of Lanzhou military command, Lan’zhou, PR China

## Abstract

**Purpose:**

Stromal-derived factor (SDF)-1 is a chemokine that recruits bone marrow-derived endothelial precursor cells (EPCs) for choroidal neovascularization (CNV) development. Angiotensin-converting enzyme (ACE) inhibitors mediate the compensatory effects of ACE and CD26/dipeptidyl peptidase IV (DPP IV), which results in the degradation and inactivation of SDF-1 in vivo. ACE inhibitors, such as imidapril, exhibit potential antiangiogenic effects on laser-induced CNV in mice. The role that this imidapril–mediated effect plays in modulating SDF-1 signals has not been defined. The present study assessed the effect of the CD26/SDF-1 signaling pathway on the inhibitory effect of imidapril in CNV development.

**Methods:**

CNV was induced in C57BL/6J mice by focally rupturing Bruch’s membrane using a 532-nm diode laser. The animals were pretreated with PBS, imidapril, diprotin-A (a DPP IV antagonist), or imidapril plus diprotin-A for 5 days before photocoagulation. Treatments were continued daily for 14 days following the laser induction. The normal control group did not undergo laser rupture or receive treatment. CD26 activity was measured using a substrate conversion assay and flow cytometry. SDF-1 levels in both the blood and the bone marrow were measured using an enzyme-linked immunosorbent assay, and the number of circulating endothelial progenitor cells (EPCs) and leukocytes was quantified. Functional analyses of circulating SDF-1 were performed using actin polymerization blood biomarker assays, and the CNV-related responses were evaluated using fluorescein angiography and isolectin-B4-labeled flatmounts.

**Results:**

Imidapril directly amplified CD26 activity and had a minor effect on the number of CD26^+^ cells in the bone marrow. However, decreased CD26 activity in the plasma was secondary to a decrease in the number of circulating CD26^+^ cells and blood leukocytes. Furthermore, imidapril increased SDF-1 concentrations in the peripheral circulation via CD26-induced degradation of SDF-1 in the bone marrow, an effect that coincided with elevated numbers of circulating EPCs. CD26-mediated SDF-1 inactivation was demonstrated by a decrease in SDF-1-induced actin polymerization in the whole blood of imidapril-treated mice. Imidapril markedly decreased angiographic leakage and CNV size. CD26 inhibition completely blocked the CD26/SDF-1 signaling pathway in vivo and reduced the antiangiogenic effect of imidapril.

**Conclusions:**

These results strongly suggest that the antiangiogenic effects of imidapril on laser-induced CNV partially involve the modulation of the CD26/SDF-1 signaling pathway.

## Introduction

Age-related macular degeneration (AMD) is a common irreversible cause of severe vision loss, including legal blindness, in the elderly population [1]. Choroidal neovascularization (CNV) is the principal cause of severe vision loss (i.e., neovascular AMD). The pathogenesis of CNV is poorly understood, and the treatment options are limited. CNV was initially believed to arise from local angiogenic events, but recent studies have suggested that bone marrow-derived cells are recruited from the circulating population and contribute to CNV formation [2-9]. Circulating endothelial precursor cells (EPCs) are derived from hematopoietic stem cells (HSCs) [10] and provide approximately 40%–50% of the vascular cells for CNV [5,6,8]. Chemokines, such as stromal-derived factor (SDF)-1, may modulate the trafficking of EPCs via specific binding to G-protein-coupled CXC receptor 4 (CXCR4) during new blood vessel formation [11].

The role of SDF-1 in CNV includes directing the EPCs to injury sites as well as the development and progression of CNV [12]. Therefore, strategies that inhibit SDF-1-driven signals should have therapeutic implications. Local SDF-1 concentrations increase vasculogenesis by increasing EPC recruitment to damaged tissues [13]. As part of the injury response, SDF-1 is upregulated in damaged choroidal and retinal tissues during ocular neovascularization, and SDF-1 may recruit stem/progenitor cells to neoangiogenic niches [11,12,14-17]. Chemotaxis assays have demonstrated that purified EPCs migrate along an SDF-1 concentration gradient in vitro [18]. Increased numbers of circulating EPCs/HSCs and lower plasma SDF-1 levels have been observed in patients with CNV [19-22]. A pathophysiological linkage between the attraction of bone marrow-derived cells to the damaged retina and low SDF-1 plasma levels may be present in the process of AMD progression [22]. Moreover, the direct blockade of SDF-1 activity in the eye reduces EPC recruitment to the CNV lesion and the EPC contribution to blood vessel formation [12]. The cleavage and inactivation of SDF-1 may play important roles in stem cell trafficking by activating molecular pathways, including protease activation, cytokine release, and chemotaxis [23-25]. We therefore examined whether the systemic modulation of SDF-1-driven signals (via protease activation to alter the release and inactivation of SDF-1) exerts a potent antiangiogenic effect in CNV.

The ectopeptidase dipeptidyl peptidase IV (DPP IV)/CD26 is a type II cell surface glycoprotein that may regulate the biologic activity of SDF-1 [23] and play a critical role in angiogenesis [26]. CD26 is a membrane-bound protease that is expressed in a variety of cells, but it is also present in a soluble form within the plasma and hematopoietic environments [27-30]. The soluble form of CD26 exerts enzymatic activity by cleaving dipeptides from the N-termini of polypeptides. CD26 activates, inactivates, or modulates the activity of various bioactive peptides, including chemokines [26,27,31]. The N-terminal region of SDF-1 is critical for receptor activation and function [32], and the truncation of this region by CD26 to form processed/truncated SDF-l (i.e., SDF-l: 3–68) inhibits the chemotactic properties of the cell and CXCR4 signaling properties [33]. The hydrolysis of SDF-1 by CD26 is important for the regulation of bone marrow-derived cell trafficking [29,34,35].

Using angiotensin-converting enzyme (ACE) inhibitors to modify the CD26/SDF-1 mechanism of EPC mobilization is a novel approach [36]. CD26 and ACE belong to the dipeptidyl peptidase family and exert their proteolytic activity in a balanced fashion to regulate the metabolism and function of peptide hormones [36]. CD26 complements or emulates the function of ACE in regulating the activation of various chemokines [37]. CD26 primarily degrades and inactivates SDF-1 in vivo in the presence of ACE inhibition [38]. ACE inhibitors may exert antiangiogenic effects in various rodent models of ocular neovascularization, including the laser-induced CNV mouse model [39-42]. Nevertheless, the role of CD26/SDF-1 in mediating the antiangiogenic effect of ACE inhibition during CNV has not been examined.

The current study provided evidence that the ACE inhibitor imidapril activates the CD26 system. Furthermore, we investigated the role of imidapril in modulating the systemic SDF-1 signaling pathway via CD26 protease-mediated degradation in a murine model of laser-induced CNV. The results demonstrated that imidapril blunts systemic SDF-1-driven signals, reducing choroidal leakage and angiogenesis in an experimental model of CNV.

## Methods

### Animals

C57BL/6 male mice (6–8-weeks old) were purchased from the Experimental Animal Center of the Fourth Military Medical University (Xi’an, China). All procedures were performed in compliance with committee-approved protocols and the Association for Vision Research and Ophthalmology Statement for the Use of Animals in Ophthalmic and Vision Research. All of the experimental protocols used in the present study were approved by the Ethics Committee for Animal Experimentation and were carried out in accordance with the Guideline for Animal Experimentation of the Fourth Military Medical University.

### Treatment protocol

The animals were pretreated with PBS, imidapril (an ACE inhibitor; Tanabe, Tianjin, China), diprotin-A (a DPP IV antagonist; GL Biochem, Shanghai, China), or imidapril plus diprotin-A for 5 days before photocoagulation. The treatments were continued daily for 14 days following laser induction. The normal control group did not undergo laser rupture or receive treatment. The mice in the drug-treatment groups received PBS (200 μl, intragastrically), imidapril (1 mg/kg/day, intragastrically), diprotin A (5 μmol twice/day, subcutaneously) or imidapril (1 mg/kg/day) plus diprotin A (5 μmol twice/day). We used this treatment protocol given that this dose of imidapril (1 mg/kg/day) by gavage was previously demonstrated to be effective for the inhibition of laser-induced CNV [42] and did not affect the blood pressure [43].

### Laser photocoagulation

Laser photocoagulation-induced rupture of Bruch’s membrane was used to induce CNV, as previously described [44]. Briefly, the animals were anesthetized via intraperitoneal injections of pentobarbital, and their pupils were dilated with topical 1% tropicamide (Santen, Osaka, Japan). Laser photocoagulation using a 532-nm diode (Viridis Twin, Quantel Medical SA, Clermont-Ferrand, France) was performed on both eyes of each mouse on day 6 following the drug treatment. Each animal received five spots per eye around the optic disc (75-μm spot size, 0.1 s duration, 100 mW), and the development of a bubble under the laser confirmed the rupture of Bruch’s membrane.

### Fluorescence-activated cell sorting analysis

Blood and bone marrow samples were freshly collected from the mice 12 days following laser-induced CNV. Circulating or bone marrow cells were identified using the nucleated cell fraction. To quantify EPCs, the number of CD34^+^/ vascular endothelial growth factor receptor 2 (VEGFR2)^+^ cells was counted in the peripheral blood. EPCs were defined by positive staining for an fluoroscein isothiocyanate (FITC)-conjugated anti-CD34 monoclonal antibody (BD PharMingen, San Diego, CA) and a phycoerythrin (PE)-conjugated anti-Flk-1 antibody (BD PharMingen), as previously described [45]. Briefly, the samples were incubated at 4°C in the dark for 30 minutes with FITC-conjugated anti-mouse CD34 and PE-conjugated anti-mouse Flk-1. After incubation, the samples were mixed with 1X lysing buffer (BD PharMingen) for 15 min at room temperature. The samples were then washed twice in phosphate buffer saline (PBS)/1% bovine serum albumin (BSA) and fixed in 4% paraformaldehyde at 4°C in dark until measured. Additionally, the number of CD26^+^ cells in the bone marrow and the peripheral blood was estimated using an FITC-conjugated anti-mouse CD26 antibody (BD PharMingen), as previously described [28]. The samples were incubated at 4°C in the dark for 30 min with FITC-conjugated anti-mouse CD26. After incubation, the samples were mixed with 1X lysing buffer (BD PharMingen) for 15 min at room temperature. The samples were then washed twice in PBS/1% BSA and fixed in 4% paraformaldehyde at 4 °C in dark until measured. Isotype-identical antibodies served as controls (BD PharMingen). The sample was analyzed on a FACScan Flow Cytometer (BD, San Jose, CA).

### Measurement of CD26 activity

Murine plasma and bone marrow extracellular fluids were collected from all groups 12 days following laser-induced CNV. CD26 activity was determined in 10-μl samples (plasma or bone marrow extracellular fluids), using the chromogenic substrate Gly-Pro-p-nitroanilide (Gly-Pro-pNA; Sigma-Aldrich, Saint Louis, MO) and according to the procedure described by Jost and colleagues [46] with minor modifications. The samples were immediately centrifuged (2000 × g; 10 min at room temperature) and each sample was pipetted in duplicates directly in a 96-well plate placed on ice. After all samples and standards (0–10 mU/ml purified DPP IV enzyme from the porcine kidney; Sigma, Deisenhofen, Germany) were pipetted, the plate was placed on a 37 °C heating block and 40-μl freshly prepared substrate solution (5 mM Gly-Pro-pNA diluted in 0.04 M HEPES buffer, pH 7.4) added in each well. The 96-well plate was now immediately placed in a microplate reader (Bio-Rad, Hercules, CA) and measurements taken at 37 °C over a period of 1 hour with a wavelength of 405 nm. Blanks (plasma+HEPES buffer) were subtracted from the measured sample values, and the DPP IV activity (U/l) was calculated using the standard calibration.

### Assessment of white blood cells in the peripheral blood

Whole blood samples were collected from the animals via a tail vein 12 days following laser-induced CNV. The total number of white blood cells (WBCs) was assessed using a Neubauer hemocytometer (Nanjing, China).

### Cytokine measurements

Murine peripheral blood samples and bone marrow were obtained from all groups following laser-induced CNV on days 0, 3, 7, and 14. The blood was centrifuged to collect the plasma, whereas the bone marrow extracellular fluids were extracted from the femurs, as previously described [47]. Samples in bone marrow were prepared for analysis by flushing the contents of 2 femurs directly into 0.4 ml sample buffer (0.1% BSA, 0.05% Tween 20 in 20 mM Trizma base, 150 mM NaCl, pH 7.3) and centrifuged. The plasma and bone marrow SDF-1 concentrations were measured using a mouse CXC ligand 12/SDF-1α enzyme-linked immunosorbent assay (ELISA) kit (R&D Systems, Shanghai, China), according to the manufacturer’s instructions. Samples in blood was obtained by centrifugation of blood at 4 °C and 2000 × g for 20 min and used for the determination of SDF-1 levels. 96-well plates were added with 50-μl of assay diluent RD1-55 (R&D systems), added with 50-μl of standard, control, or sample per well, and incubated for 2 h at room temperature on a horizontal orbital microplate shaker set at 500±50 rpm. The wells were washed three times with 400-μl of wash buffer (R&D systems) and incubated for 2 h at room temperature with 100-μl of mouse SDF-1α conjugate (R&D systems). After three washes, 200-μl of substrate solution (R&D systems) was added and incubated for 30 min at room temperature. 50-μl of stop solution stopped the reaction. A microplate reader set at 450 nm was used to determine optical density with readings at 570 nm subtracted from the results. Recombinant mouse SDF-1α sandard (R&D Systems) was used to generate a linear standard curve.

### Actin polymerization

The whole blood samples that were collected from the animals 12 days following laser-induced CNV were stimulated with 30 nM of SDF-1 (recombinant mouse CXCR12/SDF-1α; R&D Systems, Minneapolis, MN) in whole blood assay buffer (RPMI 1640, 10 mM HEPES, 0.5% FBS). The reaction was stopped by adding 1.6% formaldehyde at room temperature. Flow cytometry lysis buffer (BD PharMingen) and distilled water were added to lyse the cells. The cells were washed, fixed with formaldehyde, permeabilized with lysophosphatidylcholine (Sigma-Aldrich), and stained with FITC-phalloidin (Sigma-Aldrich). Unstimulated blood samples (blanks) were analyzed in parallel. The lymphocyte population was gated, and the median ﬂuorescence was measured using a FACScan flow cytometer (BD) [48,49].

### Fluorescein angiography and leakage grading

Fluorescein angiography (FA) was performed 13 days after laser photocoagulation. Following an intraperitoneal injection of 2.5% fluorescein sodium (Wuzhou Pharmaceutical, Guangxi, China), leakage appeared in the retina within 5 to 10 s when imaged using a modified confocal cSLO (Heidelberg Retina Angiograph; Heidelberg Engineering, Heidelberg, Germany). The digital images were taken at 3min for comparison. For the semiquantitative analyses of fluorescein leakage, the largest leakage in each eye was determined as previously described [50]. Briefly, leakage was defined as the presence of a hyperfluorescent lesion that increased in size with time during the late-phase angiogram. The leakage scores were determined by a blinded grader. The angiograms were graded as follows: grade-0 lesions, no leakage; grade-1 lesions, slight leakage; grade-2 lesions, moderate leakage; grade-3 lesions, prominent leakage.

### Volume of the choroidal neovascularization

Fourteen days following laser injury, the eyes were enucleated and ﬁxed with 4% paraformaldehyde. The cornea and lens were removed, and the eyecups were dehydrated and rehydrated through a methanol series. The eyecups were blocked with PBS that contained 1% BSA and 0.5% Triton X-100 and were stained overnight with 0.5% FITC-*Griffonia simplicifolia* isolectin-B4 (1:100; Vector Laboratories, Burlingame, CA). The retinas were removed from the eyecups, and the retinal pigment epithelium (RPE)-choroid-sclera complex was flatmounted in aqueous mounting media and coverslipped. CNV was visualized with a blue argon laser (wavelength, 488 nm), using a scanning laser confocal microscope (Olympus Corporation, Tokyo, Japan). Horizontal optical sections of the CNV were obtained at every 1-μm step from the surface to the deepest focal plane. The deepest focal plane in which the surrounding choroidal vascular network connecting to the lesion could be identified was judged to be the ﬂoor of the lesion. The area of CNV-related ﬂuorescence was measured using ImageJ (National Institutes of Health, Bethesda, MD), as described previously [51,52]. The summation of the whole fluorescent area was used as the volume of CNV.

### Statistical analysis

Statistical analyses were performed using SPSS 16.0 for Windows (Chicago, IL). All values (except the FA score) were expressed as the mean±standard deviation and were analyzed using a one-way analysis of variance. Differences between the two groups were tested post hoc using the Student–Newman–Keuls-*q* test. The FA scores were expressed as the median (range) and were analyzed using a nonparametric method (Kruskal–Wallis test) followed by the Mann–Whitney U-test. Statistical significance was defined as p<0.05.

## Results

### Imidapril activates the CD26 system, disrupting the steady-state homeostasis between CD26 activity in the bone marrow and the peripheral blood

**Figure 1:**
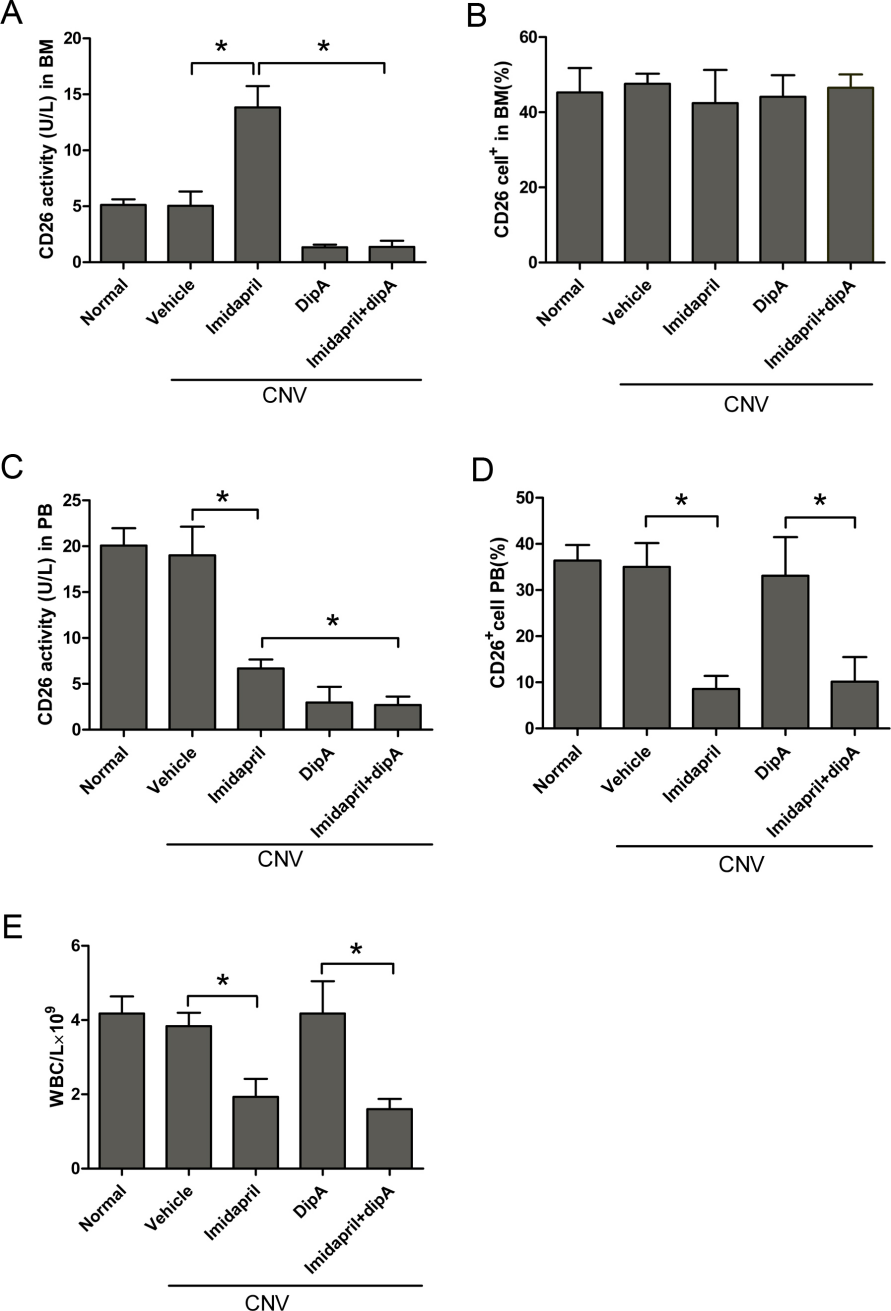
Imidapril activates the CD26 system by elevating CD26 activity in the bone marrow (BM) but not in the peripheral blood 12 days following laser-induced choroidal neovascularization. CD26 activity in the BM was assessed (**A**), and the number of CD26^+^ cells in the BM was determined (**B**). The plasma CD26 activity was measured (**C**), and the number of circulating CD26^+^ cells in the peripheral blood (PB) was determined (**D**). The number of white blood cells (WBCs) in the PB was measured (**E**). The values are expressed as the mean±standard deviation (n=5). The asterisk (*) indicates p<0.05. Dip-A represents diprotin-A. CNV represents choroidal neovascularization.

CD26 activity levels in the bone marrow supernatant and plasma, as well as the number of CD26^+^ cells in the bone marrow and circulating blood, were measured 12 days following laser-induced CNV to investigate imidapril-mediated activation of the CD26 system. Imidapril directly increased CD26 activity but did not affect the number of CD26^+^ cells in the bone marrow (Figure 1A,B). Imidapril significantly upregulated CD26 activity in the bone marrow compared with the vehicle (12.84±1.895 versus 5.04±1.289 U/l, p<0.05, Figure 1A). However, the percentage of CD26^+^ cells in the bone marrow was not significantly different between the imidapril-treated group and the other groups (p>0.05, Figure 1B). Imidapril significantly decreased plasma CD26 peptidase activity and the number of CD26^+^ cells and leukocytes in the peripheral blood (Figure 1C-E). CD26 enzymatic activity in the plasma of the imidapril-treated mice was significantly lower compared with the vehicle-treated mice (6.68±0.978 versus 19.02±3.111 U/l, p<0.05, Figure 1C). A statistically significant decrease in the percentage of circulating CD26^+^ cells was observed in the imidapril-treated group compared with the vehicle group (8.5±2.82% versus 35.0±5.18%, p<0.05, Figure 1D). CD26 activity in the plasma was reflected in the WBC. The imidapril-treated mice exhibited reduced circulating leukocyte counts compared with the vehicle-treated mice (1.93±0.485 versus 3.83±0.359×10^9^/l, p<0.05, Figure 1E). Treatment with diprotin-A completely blocked CD26 activity in the bone marrow and peripheral blood in the imidapril-treated mice, but this effect was not dependent on the altered number of CD26^+^ cells and WBCs. CD26 activity was significantly decreased by the imidapril plus diprotin-A treatment compared with imidapril treatment alone in both bone marrow (1.38±0.549 versus 12.84±1.895 U/l, p<0.05, Figure 1A) and plasma (2.70±0.905 versus 6.68±0.978 U/l, p<0.05, Figure 1C). No significant differences in the number of circulating CD26^+^ cells or WBCs were observed between the imidapril plus diprotin-A-treated and imidapril groups (p>0.05, Figure 1D-E). However, treatment with imidapril plus diprotin-A resulted in fewer circulating CD26^+^ cells (10.1±5.30% versus 33.1±8.34%, p<0.05, Figure 1D) and a lower leukocyte count (1.60±0.273 versus 4.18±0.867×10^9^/l; p<0.05, Figure 1E) compared with treatment with diprotin-A alone. These results demonstrated that imidapril effectively activated the CD26 system in a laser-induced CNV model.

### Effect of imidapril on increased stromal-derived factor-1 levels in peripheral blood due to CD26-mediated degradation of stromal-derived factor-1 in bone marrow

**Figure 2:**
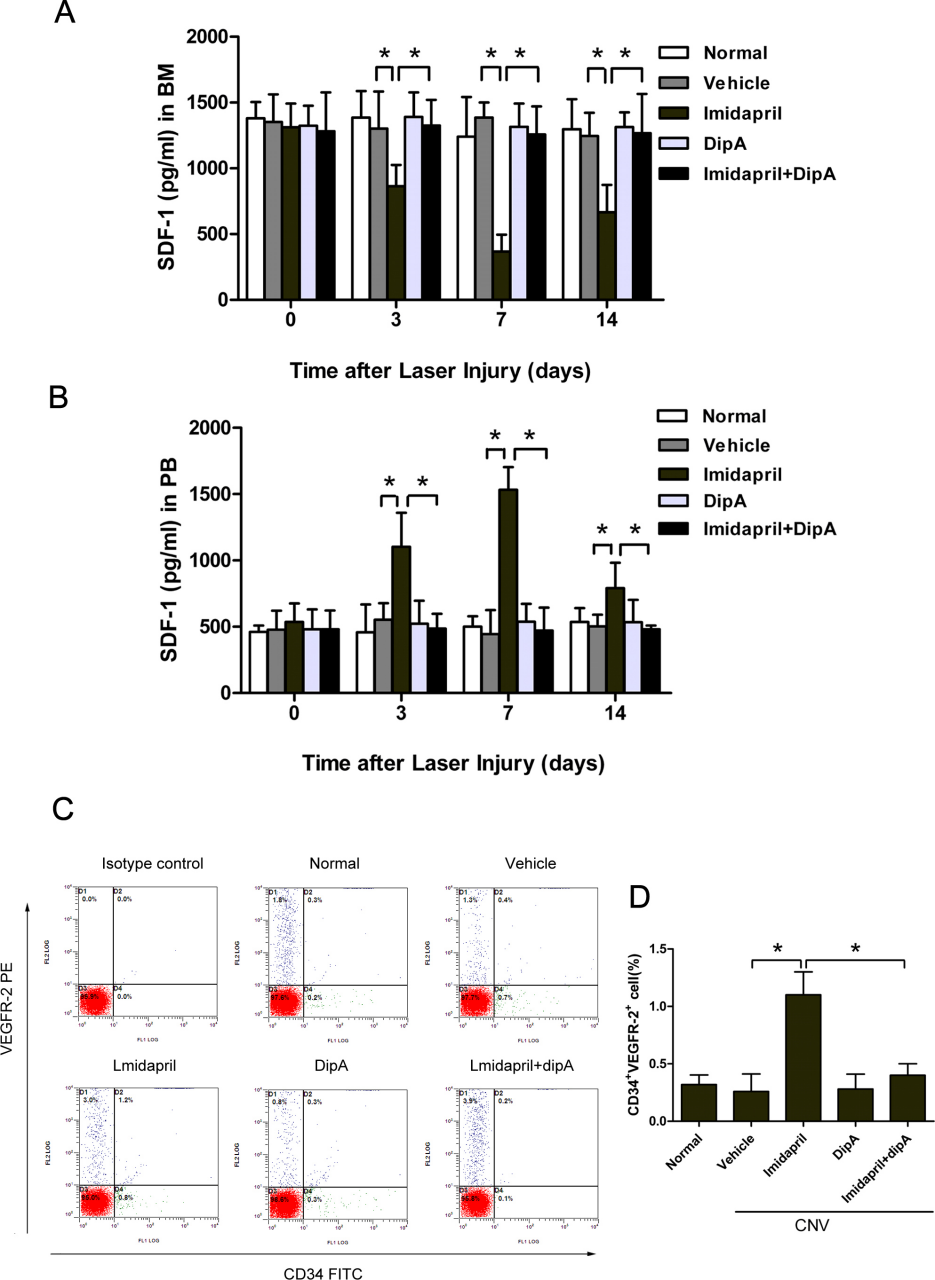
Imidapril elevates plasma stromal-derived factor-1 levels and the number of circulating endothelial precursor cells. Stromal-derived factor (SDF)-1 protein expression in the bone marrow supernatant (**A**) and the plasma (**B**) was determined using ELISA 0, 3, 7, and 14 days following laser-induced choroidal neovascularization (CNV). **C**: Representative fluorescence-activated cell sorting (FACS) data, based on which the CD34^+^/ vascular endothelial growth factor receptor 2 (VEGFR2)^+^ cells within the nucleated cell fraction of the peripheral blood from the different treatment groups were determined to be endothelial precursor cells (EPCs), are shown. **D**: The number of EPCs in the peripheral blood was determined using flow cytometry 12 days following laser-induced CNV. The values are expressed as the mean±standard deviation (n=5). The asterisk (*) indicates p<0.05. Dip-A represents diprotin-A.

Imidapril significantly activated the CD26 system in vivo. We therefore examined the effect of imidapril on the bone marrow and plasma SDF-1 levels due to CD26 proteolytic activity on days 0, 3, 7, and 14, using both ELISAs and EPC mobilization analyses 12 days following laser-induced CNV. The data (Figure 2A-D) revealed that imidapril reversed the SDF-1 gradient by altering the CD26 system, leading to EPC mobilization. SDF-1 levels in the bone marrow gradually declined in the imidapril-treated mice compared with the vehicle-treated group following laser-induced CNV (864±162 versus 1,302±281 pg/ml on day 3, p<0.05; 368±127 versus 1,386±114 pg/ml on day 7, p<0.05; and 666±207 versus 1,246±176 pg/ml on day 14, p<0.05). No differences between the SDF-1 levels were observed at day 0 (p>0.05, Figure 2A). Plasma SDF-1 levels gradually increased in the imidapril-treated mice compared with the vehicle-treated group (1,102±257 versus 552±125 pg/ml on day 3, p<0.05; 1,532±172 versus 444±181 pg/ml on day 7, p<0.05; and 790±192 versus 502±88 pg/ml on day 14, p<0.05). However, no differences between plasma SDF-1 levels were observed on day 0 (p>0.05, Figure 2B). Photocoagulation alone did not disturb the SDF-1 gradient between the bone marrow and the peripheral blood. Neither plasma nor bone marrow SDF-1 levels differed between the vehicle-treated and normal groups at any of the indicated time points (p>0.05 Figure 2 A,B). Double-positive CD34^+^/VEGFR2^+^ cells were quantified using flow cytometry in the peripheral blood to investigate whether the reversal in the SDF-1 gradient was accompanied by an increase in EPC mobilization to the peripheral blood in the imidapril-treated mice. An approximately 4.2-fold increase in the number of circulating EPCs was observed in the imidapril-treated group compared with the vehicle-treated group (1.10±0.20% versus 0.26±0.15%, p<0.05, Figure 2D). The in vivo inhibition of CD26 signaling using diprotin-A effectively corrected the reversal in the SDF-1 gradient and inhibited EPC mobilization into the peripheral blood (Figure 2A-D). The treatment with imidapril plus diprotin-A significantly increased the SDF-1 levels in the bone marrow compared with imidapril alone (1,326±195 versus 864±162 pg/ml on day 3, p<0.05; 1,258±212 versus 386±127 pg/ml on day 7, p<0.05; and 1,268±297 versus 660±207 pg/ml on day 14, p<0.05, Figure 2A), decreased plasma SDF-1 (486±111 versus1,102±257 pg/ml on day 3, p<0.05; 470±173 versus 1,532±172 pg/ml on day 7, p<0.05; and 480±29 versus 790±192 pg/ml on day 14, p<0.05; Figure 2B), and decreased the circulating EPC numbers by about 2.7-fold on day 12 (0.40±0.10% versus 1.10±0.20%, p<0.05, Figure 2D). These data demonstrated that the imidapril-activated CD26 system effectively reversed the SDF-1 gradient in laser-induced CNV.

### Imidapril negatively modulates the function of stromal-derived factor-1 via CD26-mediated degradation

**Figure 3:**
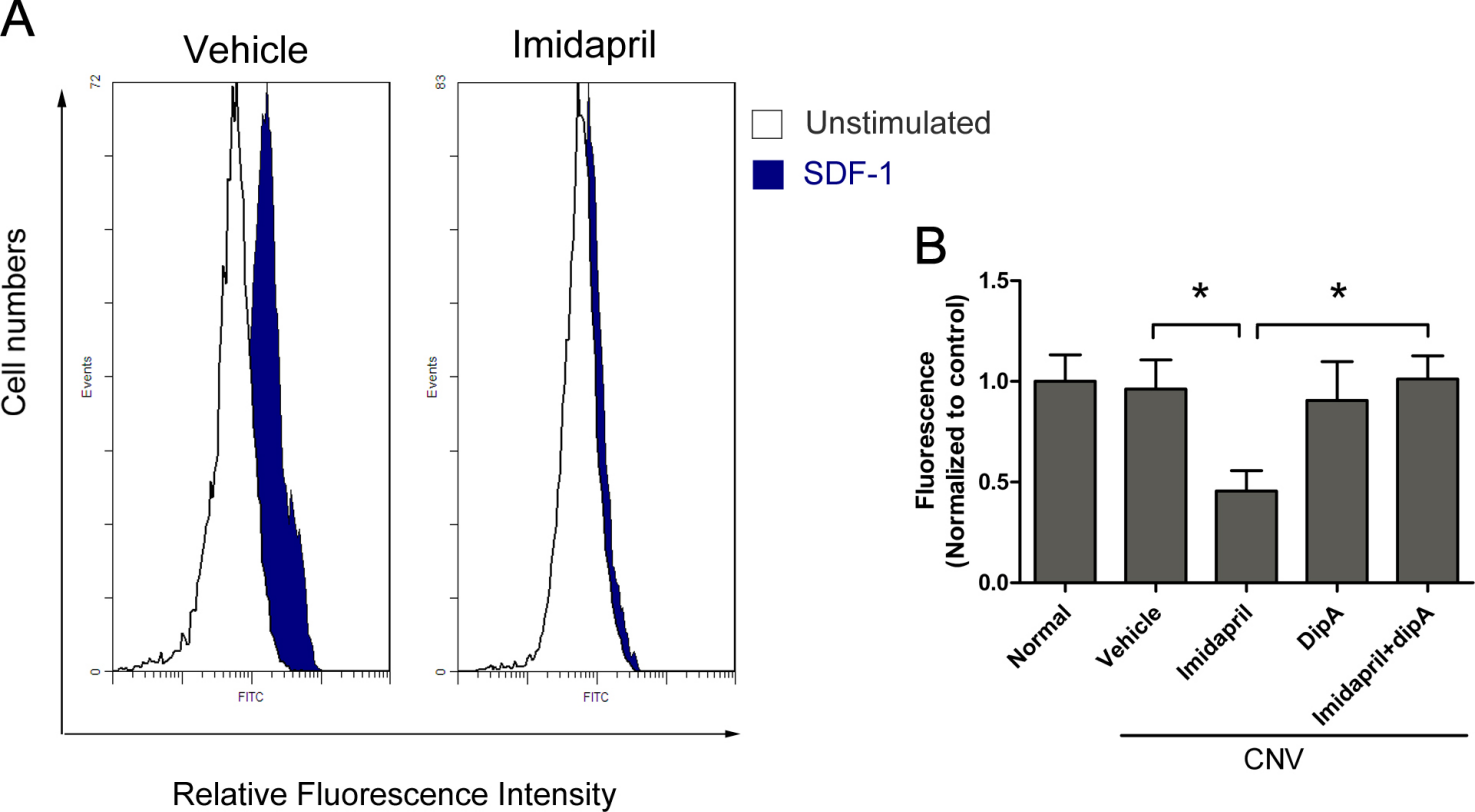
In vivo and ex vivo characterization of stromal-derived factor-1 target modification in imidapril-treated animals. Whole blood (WB) from various animals was collected 12 days following laser-induced choroidal neovascularization (CNV). The lymphocytes were either stimulated or unstimulated with exogenous SDF-1 and were analyzed using phalloidin staining and FACS analysis. A significant increase in F-actin polymerization fluorescence intensity was observed following stimulation with SDF-1 (blue peak) compared with the unstimulated (white peak) lymphocytes from the blood of the vehicle-treated mice, as illustrated by the two clearly distinct peaks (**A**: left histogram). SDF-1-stimulated (blue peak) lymphocytes from the blood of imidapril-treated mice exhibited slightly increased F-actin polymerization fluorescence compared with the unstimulated blood (white peak), as illustrated by the two overlapping peaks (**A**: right histogram). **B**: F-actin polymerization was measured following stimulation with SDF-1. Values are expressed as the mean±standard deviation (n=5). The asterisk (*) indicates p<0.05. Dip-A represents diprotin-A.

A large quantity of SDF-1 was released into the peripheral blood via CD26-mediated degradation in the imidapril-treated mice. We therefore examined whether the modulation of SDF-1 had antagonistic effects on exogenous SDF-1. The modulation of SDF-1 was investigated using an in vitro actin polymerization assay [48]. Briefly, blood from various animals was collected 12 days following laser-induced CNV. SDF-1-induced actin polymerization was notably inhibited in the imidapril-treated group (Figures 3A-B). Exogenous SDF-1-induced lymphocytes from vehicle-treated mice increased actin polymerization levels about 2.8-fold compared with unstimulated blood samples (Figure 3A). However, lymphocytes from the imidapril-treated mice exhibited a blunted response in SDF-1-induced actin polymerization compared to the unstimulated blood samples (Figure 3A). The imidapril-treated mice exhibited an approximate 2.1-fold reduction in SDF-1-induced actin polymerization compared with the vehicle-treated mice (p<0.05, Figure 3B). However, the observed decrease in actin polymerization in lymphocytes from the imidapril-treated mice was specifically inhibited by diprotin-A. The lymphocytes from the imidapril plus diprotin-A-treated mice exhibited an approximate 2.2-fold increase in SDF-1-induced actin polymerization compared with the imidapril-treated mice (p<0.05, Figure 3B). These data support the functional modification of SDF-1 by imidapril administration, which correlates with the specific cleavage of CD26 in vivo.

### Antiangiogenic effects of imidapril on choroidal neovascularization are partially dependent on CD26/stromal-derived factor-1 signaling

**Figure 4:**
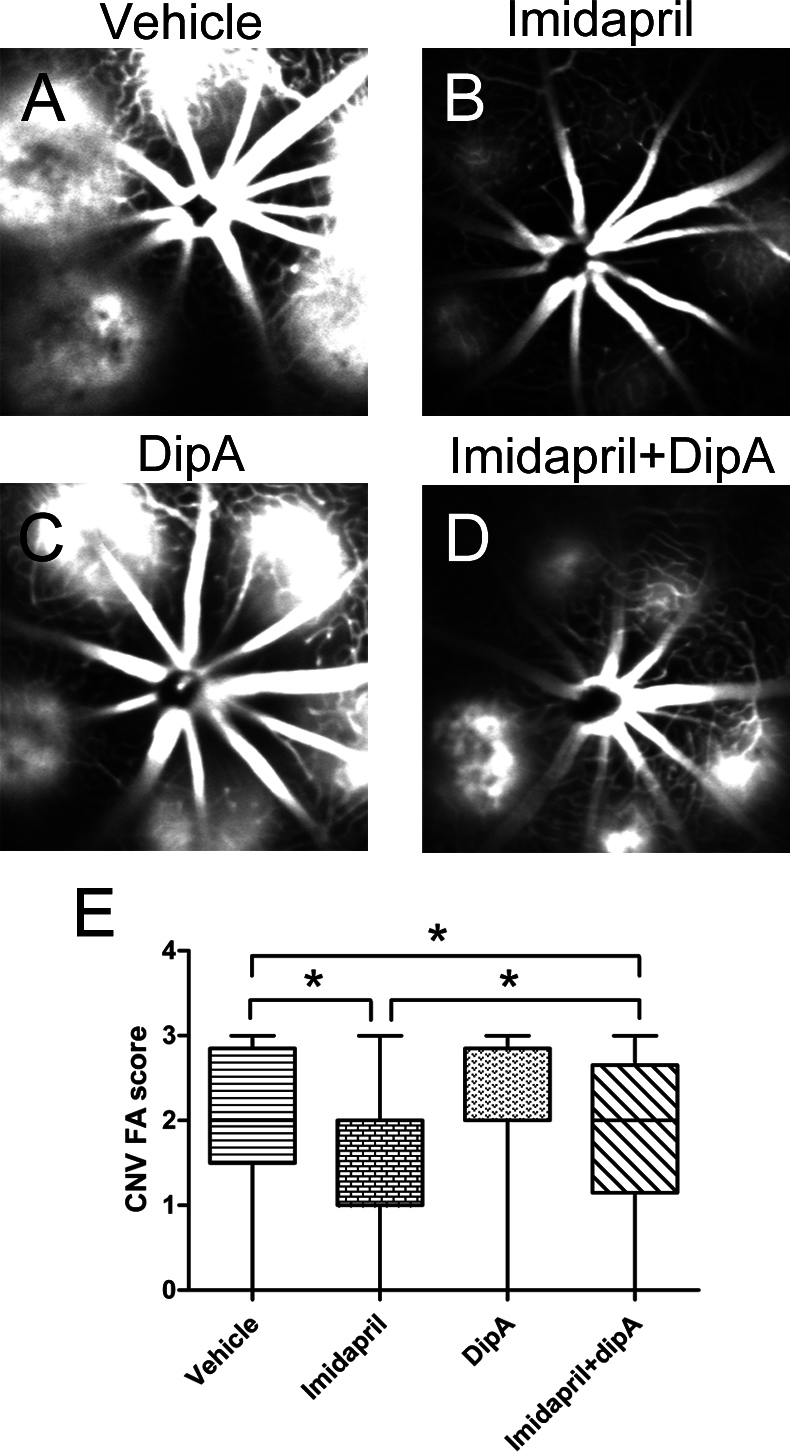
The inhibition of CD26 activity partially attenuated the imidapril-induced suppression of choroidal leakage. Representative late phase (3–4 min) fluorescein angiograms (FA) from the different treatment groups were taken 13 days following laser-induced choroidal neovascularization (CNV). A large and diffuse area of leakage was observed in vehicle-treated mice (**A**) and diprotin-A-treated mice (**C**). CNV lesions were less severe in imdapril-treated mice (**B**). In imdapril plus diprotin-A-treated mice (**D**) more moderate leakage burns were observed. In imdapril plus diprotin-A-treated mice more moderate leakage burns were observed. The results of the quantitative analyses are presented in (**E**). The values are expressed as the mean±standard deviation (n=8). The asterisk (*) indicates p<0.05. Dip-A represents diprotin-A.

**Figure 5:**
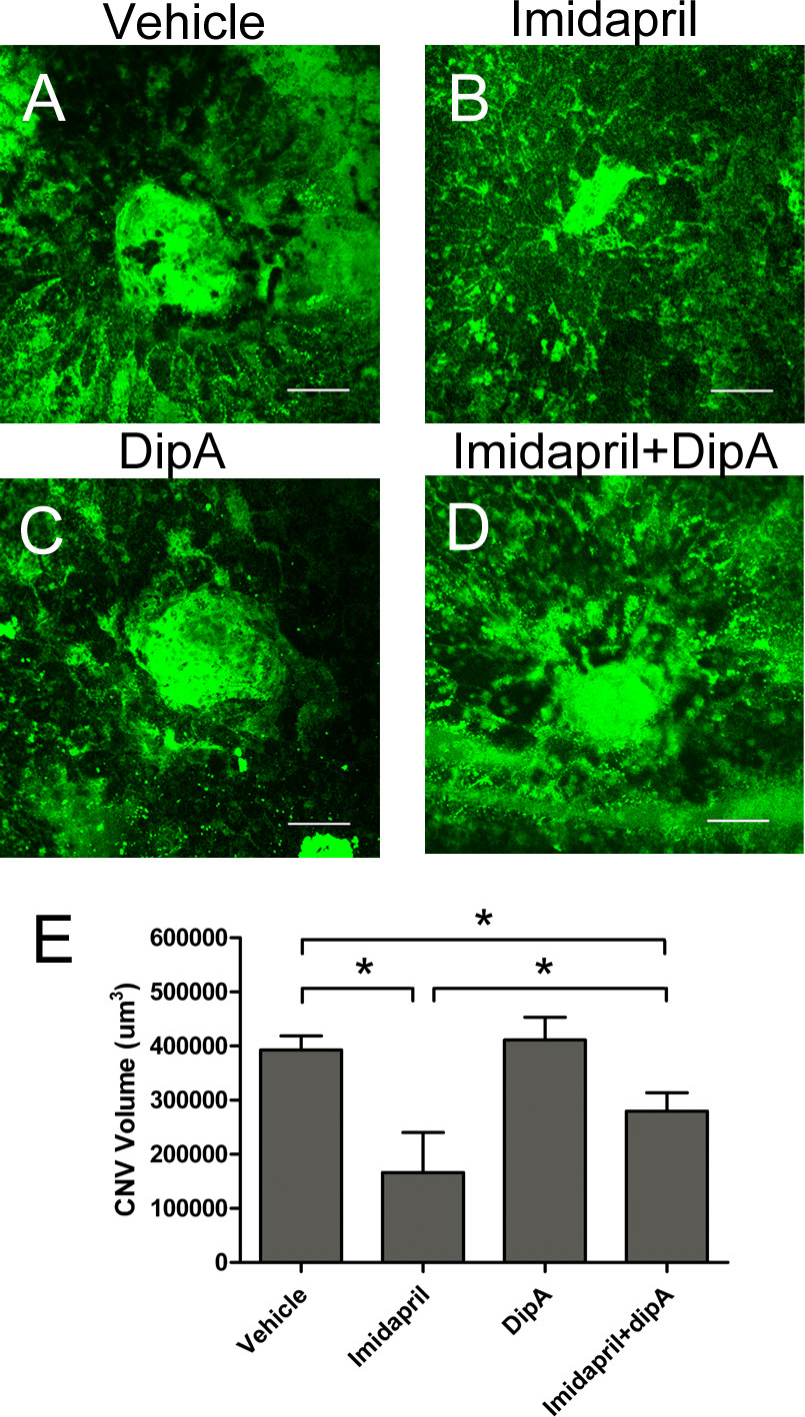
The inhibition of CD26 activity partially attenuates the imidapril-induced decrease in the choroidal neovascularization lesion volume. Isolectin B4-stained endothelial cells in choroidal flatmounts 14 days following laser injury from vehicle-treated (**A**), imidapril-treated (**B**), diprotin-A-treated (**C**), and imidapril plus diprotin-A-treated (**D**) mice are presented. The newly formed blood vessels that make up the choroidal neovascularization (CNV) were stained by isolectin-B4 immunohistochemistry and imaged by confocal microscopy, to reveal the size of the CNV lesion. The results of the quantitative analysis are illustrated in (**E**). The values are expressed as the mean±standard deviation (n=8). The asterisk (*) indicates p<0.05. Dip-A represents diprotin-A. The scale bar represents 50 μm.

Imidapril is involved in regulating the CD26-truncated modulation of SDF-1 in vivo. Therefore, the antiangiogenic effects of imidapril were analyzed using FA (Figure 4) and choroidal flatmount analysis (Figure 5) 13 and 14 days, respectively, following laser-induced CNV. Imidapril suppressed both the angiogenic lesion size and leakage in laser-induced CNV, and this effect was partially blocked by diprotin-A (Figure 4 and Figure 5). The imidapril treatment resulted in a significant inhibition of CNV leakage compared with the vehicle-treated (p<0.05) and imidapril plus diprotin-A (p<0.05) groups. However, no significant suppression of CNV was observed in the mice that were treated with diprotin-A alone (p>0.05) compared with the vehicle-treated mice (Figure 4E). The average leakage in the imidapril-treated animals was significantly less compared with imidapril plus diprotin-A treatment (p<0.05, Figure 4E). Similarly, choroidal flatmount analysis was used to quantify the CNV area. This analysis revealed a significant decrease in the volume of CNV in the imidapril-treated group (166,380±73,939 μm^3^) compared to the vehicle-treated group (392,550±26,439 μm^3^, p<0.05, Figure 5E) and the imidapril plus diprotin-A group (279,880±34,008 μm^3^, p<0.05, Figure 5E). The CNV area was less in the mice that were treated with imidapril alone compared with the imidapril plus diprotin-A-treated mice (166,380±73,939 μm^3^ versus 279,880±34,008 μm^3^, p<0.05, Figure 5E). These results and the data regarding the modulation of SDF-1 by the activated CD26 system in vivo strongly suggest that suppression of CNV leakage and growth following imidapril treatment was dependent on the CD26/SDF-1 signaling pathway.

## Discussion

The ACE inhibitor imidapril exerts potentially antiangiogenic effects on laser-induced CNV by regulating the rennin-angiotensin system [42]. However, very little is known that systemic SDF-1 signals are modulated by CD26 peptidase activity following ACE inhibition [36]. SDF-1 overexpression in damaged tissues enhances EPC recruitment from peripheral blood and induces neovascularization [11]. One potential strategy for limiting the role of EPCs in pathological neovascularization relies on the interference of SDF-1 signals in mediating CNV [12]. Strong experimental evidence has suggested that the N-terminal truncation of SDF-1 by CD26 proteases modulates SDF-1 action in vivo [53]. This truncation may have profound implications on this chemokine’s regulation of biologic activities. We therefore investigated whether the imidapril-mediated suppression of laser-induced CNV development involves the modulation of the SDF-1 signaling pathway via CD26 enzyme activity. The primary results that were observed are as follows: (i) imidapril administration activated the CD26 system in a laser-induced CNV model, disrupting the steady-state homeostasis between CD26 activity in the bone marrow and the peripheral blood; (ii) this disruption altered the gradient and function of SDF-1; (iii) imidapril reduced choroidal leakage and angiogenesis via a mechanism that was partially dependent upon the specific cleavage of CD26, which blunted the systemic SDF-1-driven signals during CNV development.

Imidapril-induced activation of the CD26 system in bone marrow and peripheral blood during experimental CNV may be part of a compensatory regulatory mechanism. ACE and CD26 exert their proteolytic activity in a balanced fashion, and ACE inhibition may exert a compensatory regulatory effect on CD26 [37,54]. Imidapril specifically activates CD26 inside of the bone marrow microenvironment to amplify CD26 activity. Meanwhile, imidapril significantly decreases CD26 activity in the plasma. ACE inhibitors induce leukopenia in humans and mice [38,55-58], and the leukocyte population is the most abundant source of CD26^+^ cells in peripheral blood [59,60]. Changes in plasma CD26 levels reflect changes in the levels of this enzyme that are released by the leukocyte population [61], and the reduced CD26 activity levels in the plasma may be secondary to the diminished number of circulating CD26^+^ cells [62]. Consistent with our results, the ACE inhibitor enalapril was reported to significantly disrupt the steady-state homeostasis of CD26 activity between the bone marrow and peripheral blood in a rodent hind limb ischemic model [38]. Moreover, the effect of imidapril on the CD26 system in vivo is significantly blocked by diprotin-A, further supporting a regulatory role of imidapril in the CD26 system. A disruption in the proteolytic balance leads to the cleavage and inactivation of molecules that are essential for the retention of hematopoietic stem and progenitor cells (HSPCs) within their niche [63]. Therefore, our study investigated whether the striking imidapril-induced changes in the CD26 proteolytic balance could effectively modulate the level and function of SDF-1 during the pathological process of laser-induced CNV.

Our study demonstrated that imidapril reversed the SDF-1 gradient via CD26 degradation of SDF-1 in the bone marrow, which may have altered the kinetics of SDF-1 during angiogenesis in the laser-induced CNV model. SDF-1 is anchored to the membrane of stromal cells, endothelial cells, or the extracellular matrix by its specific binding to HSPCs under steady-state conditions [24]. The degradation of SDF-1 by CD26 proteolytic enzymes increases SDF-1 plasma levels, and this increase promotes EPC mobilization from the bone marrow to the peripheral blood [38,64]. Conversely, increased SDF-1 concentrations in the bone marrow are caused by decreased CD26 activity, which greatly increases the transplantation efficiency of CD26^+^ stem/progenitor cells [65]. A rapid increase in SDF-1 was observed upon laser lesioning in a rodent model of CNV [15,16]. SDF-1 acts as a signaling mediator that guides the migration of bone marrow-derived cells along an SDF-1 gradient to the sites of injury in CNV [16,66,67]. The accumulation of these cells in the RPE/Bruch’s membrane/choriocapillaris complex contributes to CNV formation [12,16]. If an SDF-1 gradient is established, bone marrow-derived cell trafficking is more efficient. Conversely, if this gradient is blocked, then the homing efficiency of the cells is reduced to negligible levels. A previous study by our group demonstrated that SDF-1 expression in the laser-induced lesion adjacent to the RPE continuously increased and peaked in concert with increasing numbers of bone marrow-derived cells infiltrating the CNV [16]. A mobilizing effect that was initiated by reversing the SDF-1 gradient was observed following the administration of imidapril. This chemokine gradient may alter the kinetics of SDF-1 during angiogenesis in the CNV model and attenuate EPC homing to the sites of injury. The study by Lee E et al. [49] also confirmed that inhibition of the SDF-1/CXCR4 axis by systemic delivery of AMD3100 induces EPC mobilization, disrupting homing of the cells along an SDF-1 gradient to the vascular area of the lesion.

The specific cleavage of SDF-1 by the CD26 enzyme in vivo alters the chemotactic behavior of SDF-1 [53]. In the present study, a large quantity of SDF-1 was released into the peripheral blood following imidapril administration, and a blunted SDF-1-induced actin polymerization response was observed. This phenomenon represents the in vivo and ex vivo characterization of the modification of SDF-1 signaling by CD26-mediated degradation [34,68]. The N-terminus of SDF-1 is important for CXCR4 binding and activation. The N-terminal domain of SDF-1 (full length: 1–68) is efficiently processed by CD26 proteases, yielding a truncated form (3–68). This truncated form of SDF-1 exerts antagonistic effects that are opposed to those of full-length SDF-1 both in vivo and in vitro [53,69,70] and inhibits both CXCR4 signaling and lymphocyte chemotactic activity [33,71]. Our data regarding the specific blockade effect of diprotin-A supports the role of imidapril in modulating the CD26/SDF-1 signaling pathway. No intact SDF-1 was recovered following CD26 treatment, and no SDF-1 truncation occurred in the absence of CD26, suggesting the specificity of this cleavage in vivo [33,69]. Indeed, previous studies have indicated that SDF-1 signaling induces cytoskeletal reorganization, potentially leading to a change in HSPC motility. The magnitude of the SDF-1-induced actin polymerization response correlates positively with the migratory capacity of primary human HSCs [72]. These published studies and our data suggest that modulating SDF-1 by CD26 degradation also alters the migration of EPCs along the SDF-1 gradient to the vascular angiogenic sites.

Evidence for the central role of SDF-1 in exerting a more potent pro-angiogenic effect has prompted investigators to examine the effect of manipulating SDF-1 signaling in a therapeutic setting. Indeed, favoring revascularization by augmenting the effects of SDF-1 should be beneficial for cardiovascular diseases [73], whereas ocular diseases, such as CNV, in which blood vessel formation is detrimental, could benefit from an efficient SDF-1 blockade [12,14]. More approaches that involve systemic SDF-1 interference are likely to be developed, whether in the context of microvascular or macrovascular diseases. Due to the short half-life of SDF-1 protein in the circulation, one research group has employed genetic modification strategies by which to continuously express SDF-1 in situ, leading to enhanced angiogenesis and myocardial repair [74]. To circumvent rapid SDF-1 degradation by proteolytic enzymes in vivo, several research groups have developed strategies by which to construct a protease-resistant SDF-1 [75] or to inhibit CD26 [76]. As the stability of SDF-1 is greatly enhanced in this model, greater numbers of stem cells are recruited to the site of myocardial infarction, improving heart function. These new findings encourage the utilization of CD26/SDF-1 pathway modulations to reduce the recruitment of EPCs to injury sites and to inhibit angiogenesis in microvascular diseases. Nagai et al. demonstrated that ACE inhibition using the systemic administration of imidapril exerts antiangiogenic effects in a laser-induced CNV model [42]. These authors suggested that the benefits of imidapril in CNV treatment were primarily mediated by regulating the rennin-angiotensin system; whether the antiangiogenic effects of imidapril are involved in a CD26/SDF-1 pathway has not been determined. Our study revealed that the process of SDF-1 cleavage by CD26 may be a novel regulatory mechanism for the suppressive effect of imidapril on CNV.

One limitation of the present study was that our animal model of laser-induced CNV did not evaluate the reduction in the number of EPCs within the lesion because we used C57BL/6 wild-type rather than C57BL/6 green fluorescent protein chimeric mice. However, the animal model that was used in the present study effectively avoided the analysis of SDF-1 and EPC dynamics in a physiologically abnormal state. Chimeric mice must undergo irradiation to permit acceptance of the transplanted bone marrow and to create chimera [77]. The transplantation approach enriches HSCs, which is not reflective of normal bone marrow homeostasis [78], and the process of creating chimerism may elevate circulating SDF-1 levels and EPC mobilization [79]. Moreover, total body irradiation at a level that kills all the hematopoietic cells has deleterious effects on endothelial cells and triggers radiation retinopathy [17,80].

In conclusion, our data provide evidence that the antiangiogenic effects of imidapril on laser-induced CNV are partially dependent on its modulation of the CD26/SDF-1 signaling pathway. The mechanism of imidapril action may include the control of the CD26 protease cleavage events that directly affect the SDF-1 gradient and chemotactic function. An improved understanding of the mechanism of action of ACE inhibitors could lead to novel insights into therapeutic treatments for AMD.
